# Accuracy of corticospinal tract reconstruction: DTI-based deterministic versus probabilistic tractography with intraoperative validation

**DOI:** 10.3389/fonc.2026.1790772

**Published:** 2026-05-15

**Authors:** Roberto Altieri, Lorenzo Ugga, Ferdinando Caranci, Ciro De Luca, Giuseppe La Rocca, Daniela Pacella, Manlio Barbarisi

**Affiliations:** 1 Multidisciplinary Department of Medical-Surgical and Dental Specialties, University of Campania “Luigi Vanvitelli”, Naples, Italy; 2 Department of Advanced Medical and Surgical Sciences, University of Campania “Luigi Vanvitelli”, Naples, Italy; 3 Department of Precision Medicine, University of Campania “Luigi Vanvitelli”, Naples, Italy; 4 Laboratory of Morphology of Neuronal Network, Department of Public Medicine, University of Campania “Luigi Vanvitelli”, Naples, Italy; 5 Institute of Neurosurgery, Fondazione Policlinico Universitario A. Gemelli IRCCS, Catholic University, Rome, Italy; 6 Department of Public Health, University of Naples “Federico II”, Naples, Italy

**Keywords:** brain mapping, diffusion tensor imaging, direct electrical stimulation, probabilistic tractography, white matter

## Abstract

**Introduction:**

The precise preoperative mapping of the corticospinal tract (CST) is crucial for preserving motor integrity during brain tumor resection. While Diffusion Tensor Imaging (DTI) is widely utilized, the comparative accuracy of DTI-based deterministic versus probabilistic tractography algorithms, validated against intraoperative direct electrical stimulation (DES), remains a critical area of investigation.

**Methods:**

We retrospectively analyzed a single-center experience about patients operated on for brain tumors involving the motor areas between July 2024 and December 2025. CST reconstructions were generated using Medtronic StealthStation S8 software. Statistical analysis was applied to determine the most reliable reconstruction method, and the distance measured by each DTI technique (mm) was compared to the stimulation threshold (mA), assuming a 1:1 ratio (1mA = 1 mm).

**Results:**

40 patients underwent surgical resection and 8 patients, (mean age 58.33) meet the inclusion criteria. 30 stimulation sites were analyzed. The mean stimulation threshold was 9.83 ± 5.49 mA (range: 5–20 mA). The probabilistic tractography reached a mean distance of 12.01 ± 8.92 mm (range: 1–30.1 mm), while the DTI-based deterministic tractography showed a mean distance of 27.71 ± 17.14 mm (range: 8–71.4 mm). Paired mean difference between DES threshold and probabilistic tractography was not significant (mean difference: -2.2, 95% CI [-5.9; 1.6], p=0.24), while it was significant between DES threshold and DTI-based deterministic reconstruction (mean difference: -17.9, 95% CI [-24.9; -10.9], p<0.001) revealing that this method significantly overestimated the distances. Probabilistic tractography also held lower RMSE against the gold standard compared to deterministic (respectively 10.1 vs 25.7).

**Conclusions:**

Integrating probabilistic tractography with DES enhances the surgical safety profile for tumors involving the motor pathway. The quantitative reliability of this combined approach allows for optimized maximal-safe resection while minimizing postoperative motor deficits.

## Introduction

In the management of intrinsic brain tumors, it is well established that the extent of resection (EOR) is a primary predictor of overall survival and progression-free survival (1–6). However, the surgical benefit of maximal resection must be carefully balanced against the preservation of neurological function (7–11). Maintaining a high Karnofsky Performance Status (KPS) is essential, as postoperative deficits can preclude patients from accessing vital adjuvant therapies, such as radiotherapy and chemotherapy (12).

Among the various locations, tumors involving the motor cortex and descending pathways represent one of the most significant challenges in neurosurgery. The proximity of these lesions to “eloquent” areas requires meticulous preoperative planning to maximize tumor removal while minimizing the risk of permanent motor impairment.

Motor functions are not merely the product of localized cortical activity; rather, they depend on the complex integration of multisensory and premotor information processed across multiple cortical and subcortical levels. This orchestration is mediated by a sophisticated framework of white matter connectivity, including a short-range integration via intragyral U-fibers; intrahemispheric associations facilitated by the Fronto-Aslant Tract (FAT), the Fronto-striatal Tract, the Superior Longitudinal Fasciculus (SLF), and the Inferior Fronto-Occipital Fasciculus (IFOF); interhemispheric communication mediated through the Corpus Callosum (13). Despite the growing evidence that preserving the primary motor strip (M1) and the corticospinal tract (CST) is insufficient to guarantee the full preservation of complex movement, their identification remains mandatory in neurosurgical and clinical contexts. These structures represent the obligate final common pathway, the definitive output through which all integrated motor commands are translated into physical action.

In recent years, non-invasive imaging techniques, including functional MRI (fMRI) and Diffusion Tensor Imaging (DTI) tractography, have become indispensable tools for the neurosurgical armamentarium. Modern neuronavigation systems have further advanced these capabilities by integrating sophisticated software for the reconstruction of white matter tracts. Specifically, neurosurgeons can now choose between two primary reconstruction methods:

1. Deterministic algorithms, which follow the path of least resistance based on a fixed vector.
2. Probabilistic algorithms, which account for uncertainty and branching, potentially offering a more nuanced view of the subcortical anatomy (14).

Diffusion MRI tractography depends on both the signal representation model and the tracking algorithm. In clinical practice, deterministic tractography is most commonly coupled with diffusion tensor imaging (DTI), in which one principal diffusion direction is estimated per voxel. By contrast, probabilistic tractography generally relies on higher-order models, such as constrained spherical deconvolution (CSD), which estimate orientation distributions and can better represent complex intravoxel fiber configurations, including crossing fibers.

Consensus remains limited because deterministic and probabilistic approaches offer different trade-offs: deterministic reconstructions are generally more reproducible, computationally efficient, and easier to interpret in routine clinical settings, whereas probabilistic approaches are more sensitive to complex fiber configurations and peritumoral distortion, but may generate spurious streamlines and depend more strongly on user-defined tracking parameters. The “gold standard” for identifying eloquent structures remains intraoperative neurophysiological monitoring (IONM), including cortical and subcortical mapping with direct electrical stimulation (DES) (15, 16).

The present study aims to evaluate and compare the accuracy of deterministic versus probabilistic tractography by correlating preoperative reconstructions with intraoperative neurophysiological findings. By identifying which algorithm more closely aligns with real-time mapping, we seek to optimize preoperative planning and enhance the safety of motor area tumor resections.

## Materials and methods

This retrospective single-center study has been conducted at the Neurosurgical Unit of the University of Campania “Luigi Vanvitelli”. All patients provided informed consent for the use and publication of their anonymized data. Medical records of patients operated on for brain tumors between July 2024 and December 2025 were reviewed.

Inclusion criteria included: (I) age >18 years; (II) radiological diagnosis of a brain tumor involving the central gyri or the corticospinal tract (CST); and (III) complete preoperative neuroradiological assessment, including 3D contrast-enhanced T1-weighted and FLAIR-weighted sequences, and DTI.

Imaging was collected on a 1.5 Tesla MRI scanner (Aera, Siemens Healthineers, Erlangen, Germany) with 20 channel head coil. DTI data were obtained with 2 b=0 volumes and 32 diffusion directions using an EPI sequence (TR = 8900 ms, TE = 88 ms, section thickness = 2.5 mm, FOV = 245 × 245 mm, b=1000s/mm2). The EPI phase-encoding direction was anterior-posterior.

CST reconstructions were generated using the Medtronic StealthStation S8 fiber-tracking software (Minneapolis, MN, USA), with ROI placement following the methodology described by Feconja et al. (17–19). The same ROIs, positioned as previously specified, were applied for both reconstruction techniques. Using this approach, the tracts were successfully reconstructed with both methods in 6 of 8 patients. In the remaining 2 of 8 cases, minor adjustments of selected ROIs were required to obtain tract reconstruction with the deterministic method.

Total Intravenous Anesthesia (TIVA) was standard. Craniotomy size was tailored to minimize brain shift while ensuring adequate brain mapping. Intraoperative NeuroMonitoring (IONM) included continuous EEG, free-running EMG (ISIS-IOM, Inomed), SSEP, and motor mapping (cortical/subcortical) via MEP monitoring. Stimulation-induced seizures were managed with cold saline irrigation or intravenous levetiracetam (1 g).

**Figure 1:**
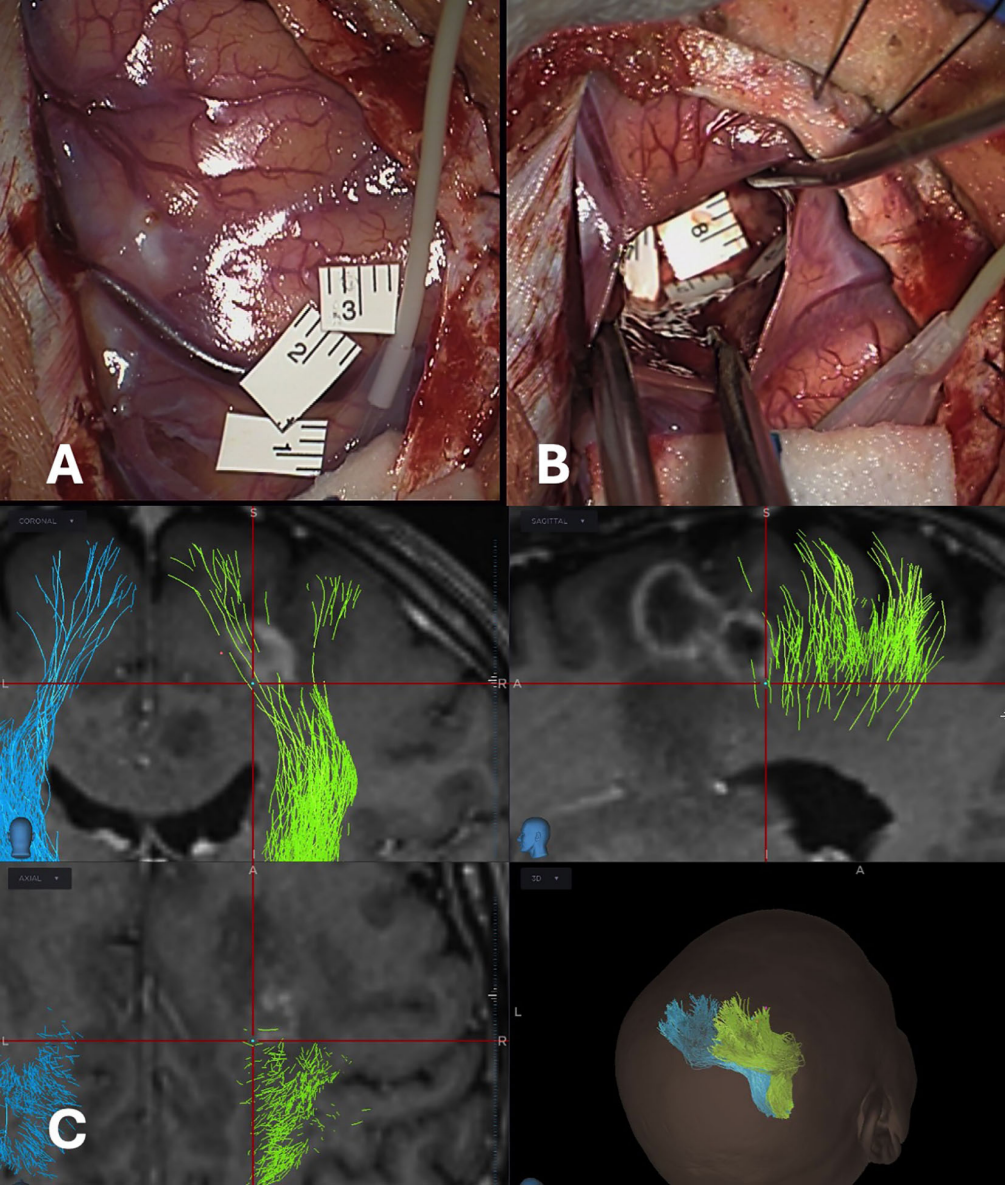
Panel **(A, B)** show, respectively, cortical and subcortical mapping of a right rolandic GBM. In Panel **(C)**, each DES-positive site is localized with a digital tag using neuronavigation.

**Figure 2:**
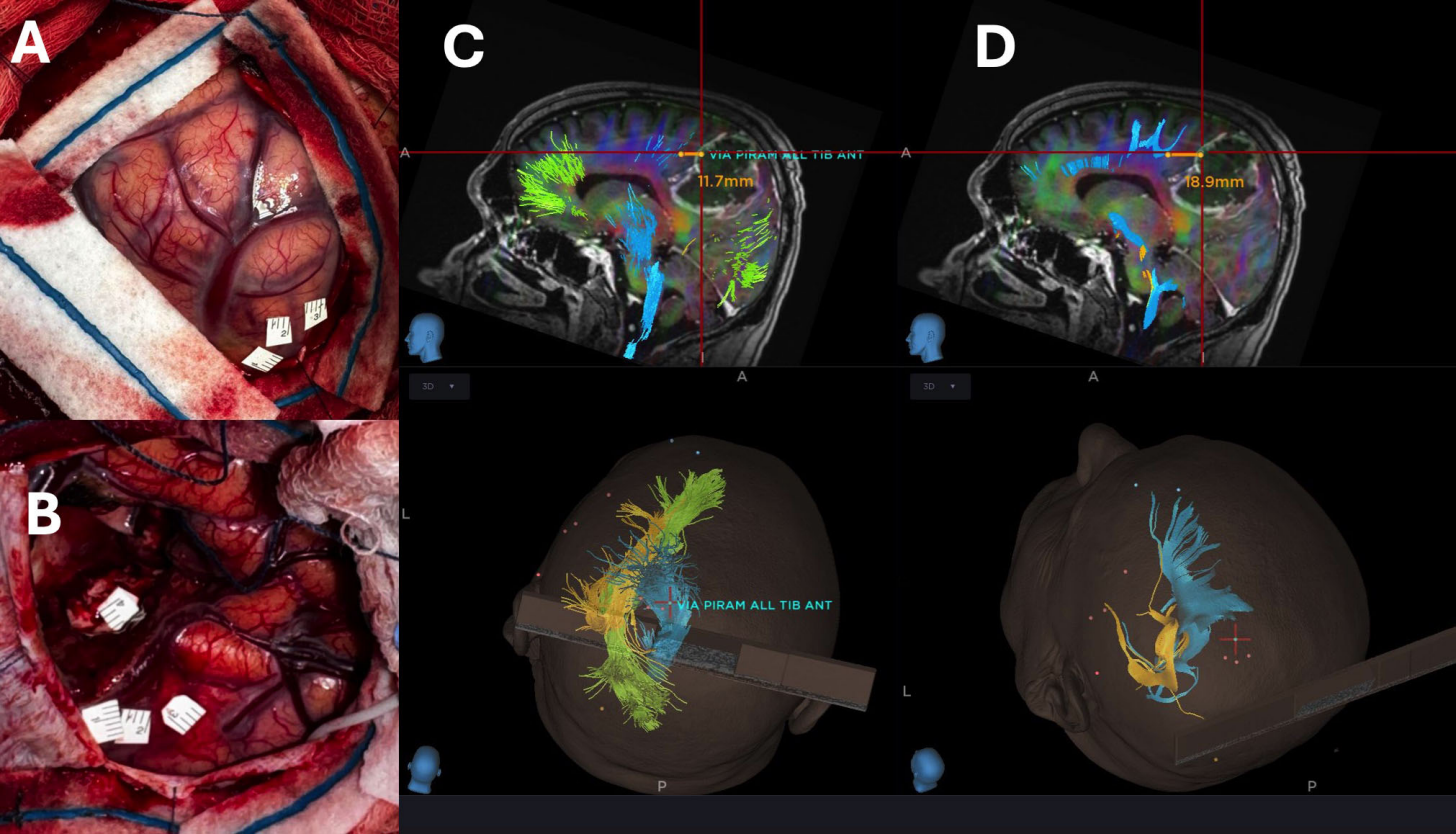
**(A, B)** show, respectively, cortical and subcortical mapping of a left rolandic GBM. The distance between the CST and each tag was subsequently measured using both Probabilistic **(C)** and Deterministic DTI reconstructions **(D)**.

We performed brain mapping using a monopolar high-frequency stimulation paradigm consisting of trains of 5 pulses with an interstimulus interval (ISI) of 4 ms (corresponding to 250 Hz) and a train duration of 500 ms. This approach was selected because it provides greater sensitivity in detecting functional sites (particularly during subcortical mapping) and allows quantitative estimation of the distance from critical subcortical tracts based on the stimulation intensity required to elicit a motor response (approximately 1 mA ≃ 1 mm) (20, 21). Brain mapping started at 2.5 mA, increasing up to 20 mA. Intraoperative brain mapping was routinely performed in all cases by the same dedicated team of neurosurgeons and neurophysiology technicians, all specifically trained to work in synergy within the neuro-oncological setting to ensure reliable real-time data interpretation. A positive response was defined as three consecutive positive feedbacks following Direct Electrical Stimulation (DES). Each DES-positive site was localized using the neuronavigation system by placing a digital tag (**Figure 1**). After intraoperative tagging of each DES-positive site, the Euclidean distance between each tag and the nearest streamline of the preoperatively reconstructed CST was measured for both the deterministic and the probabilistic tractography datasets (**Figure 2**). Tract reconstructions were performed in the preoperative phase. In the postoperative analysis, both probabilistic and deterministic reconstructions were evaluated in a blinded fashion with respect to the intraoperative mapping results.

### Statistical analysis

Data are summarized using mean and standard deviation. To determine the most reliable reconstruction method, the distance measured by each DTI technique (mm) was compared to the stimulation threshold (mA), assuming a 1:1 ratio (1mA = 1 mm) (22, 23). The significance of the paired mean difference between each method and the gold standard was assessed using univariable mixed-effect linear regressions adding patient’s ID as a random effect. These differences – i.e. the coefficient estimates - are reported alongside the corresponding 95% confidence intervals (C.I.). Given that measures were performed on the same 8 patients, to quantify the relationship between DTI reconstructions and DES measurements taking into account the patients’ individual variability, partial correlations were calculated, estimating the effects from the residuals of univariable linear mixed-effect models having patient ID as random effect. The reliability of DTI-based deterministic versus probabilistic methods was assessed by calculating the Root Mean Squared Error (RMSE). Additionally, Bland-Altman plots were computed to investigate the agreement between DTI-based deterministic or probabilistic methods and DES thresholds. All analyses were conducted using R Statistical Software version 4.4.0. A two-tailed p-value < 0.05 was considered statistically significant.

## Results

**Figure 3:**
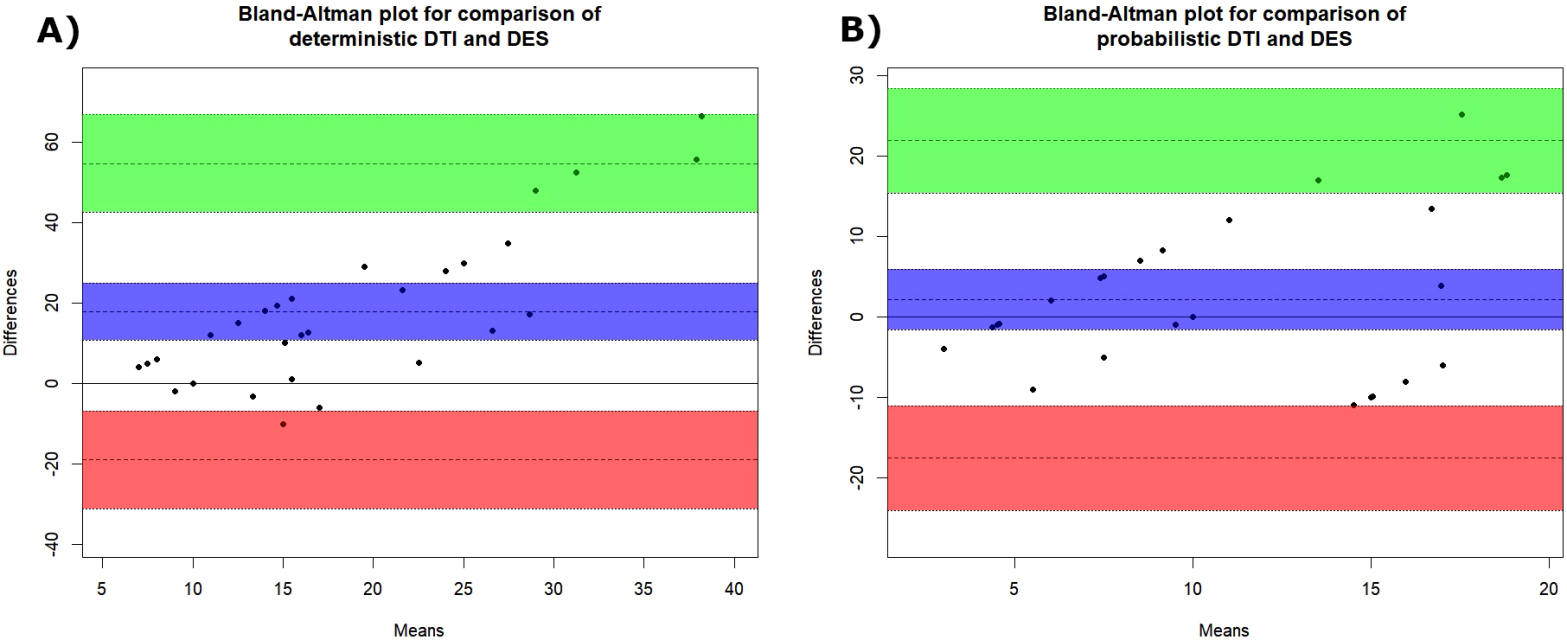
Bland-Altman plots of the agreement between deterministic DTI and DES thresholds **(A)** and between probabilistic DTI and DES thresholds **(B)**. The line surrounded by purple confidence bands represent bias line with 95% confidence intervals, the line surrounded by green confidence bands represents the upper limit of agreement with 95% confidence interval, while the line surrounded by red confidence bands represents the lower limit of agreement with 95% confidence interval.

In the selected period 40 patients underwent surgical resection for brain tumors at our institution. Following the application of strict inclusion criteria, 8 patients, all males; mean age 58.33 years old) were included in the study. KPS was 100 for 7/8 (87.5%), while the remaining patient presented with a KPS of 80. The histological diagnoses consisted of glioblastoma (GBM) in 5 cases and brain metastases in 3 cases. A total of 30 stimulation sites were analyzed to compare DES thresholds with distance measurements from probabilistic and deterministic reconstructions. The mean stimulation threshold was 9.83 ± 5.49 mA (range: 5–20 mA). Under the assumed 1:1 ratio (1 mA = 1 mm), the probabilistic tractography reached a mean distance of 12.01 ± 8.92 mm (range: 1–30.1 mm), while the deterministic reconstructions showed a much greater mean distance and variability, equal to 27.71 ± 17.14 mm (range: 8–71.4 mm). Mean difference between DES threshold and probabilistic tractography was not statistically significant (beta: -2.2, 95% C.I. [-5.6; 1.2], p=0.24), while it was significant between the DES threshold and deterministic reconstructions (beta: -17.9, 95% C.I. [-24.4; -11.8], p<0.001) revealing that this method significantly overestimated the distances. Probabilistic tractography also held lower RMSE against the gold standard compared to deterministic reconstructions (respectively 10.1 vs 25.7). Partial correlations between DTI-based deterministic or probabilistic tractography and DES thresholds revealed no significant linear relationship (respectively r=0.05, p=0.786 and r= -0.05 p=0.819). **Figure 3** displays the Bland-Altman plots of the agreement between DTI-based deterministic tractography and DES thresholds (panel A) and between probabilistic tractography and DES thresholds (panel B). The visual inspection of the plots reveals that both methods have nearly all observations within the limits of agreement but confirms a better agreement between probabilistic tractography and DES, since the DTI-based deterministic tractography plot shows a trend of increasing bias as the mean increases, resulting in extremely large measures. In fact, while the probabilistic method reveals no significant bias (p=0.244) there is a significant bias for the deterministic (p<0.001).

5 patients received a Gross Total Resection (2 GBM and 3 metastases en-bloc removed), 3 patient received a near total resection (GBM). The patient with a preoperative KPS of 80 was the only individual in the cohort to experience post-operative clinical worsening, specifically characterized by an exacerbation of their pre-existing hemiparesis (GBM). It is important to emphasize that all intraoperative surgical decisions were based exclusively on brain mapping. Therefore there is no direct relationship between clinical outcomes and any potential mismatch between DTI-based deterministic and probabilistic tractography, as the DTI data did not influence the EOR or the surgical trajectory.

## Discussion

The present study provides intraoperative validation of diffusion tensor imaging-based CST reconstruction, directly comparing deterministic and probabilistic tractography against the current functional gold standard, namely DES. Our findings suggest that probabilistic tractography more accurately reflects the true functional-anatomical relationship of the CST in patients harboring tumors involving motor pathways, whereas deterministic tractography tends to significantly overestimate the distance between stimulation-positive sites and the CST.

Maximizing extent of resection while preserving neurological function remains a central challenge in the surgical management of intrinsic brain tumors, particularly those involving eloquent motor regions. Although advances in intraoperative neurophysiological monitoring have dramatically improved surgical safety, preoperative planning still relies heavily on imaging-based reconstructions of white matter tracts. In this context, the reliability of tractography algorithms is of paramount importance, as inaccurate reconstructions may lead either to overly conservative resections or to unexpected postoperative deficits (24).

Our results demonstrate a close agreement between probabilistic tractography-derived distances and DES thresholds, assuming the widely accepted 1 mA = 1 mm relationship for subcortical motor mapping. The lack of a statistically significant difference between probabilistic distances and stimulation thresholds along with a lower overall measurement error supports the notion that probabilistic algorithms provide a more faithful representation of the CST course *in vivo*. In contrast, deterministic tractography showed a marked and statistically significant overestimation of CST distance, with poor correlation to DES findings. This discrepancy is clinically relevant, as it may falsely reassure the surgeon regarding the safety margin from motor pathways.

The observed differences can be explained by the fundamental methodological distinctions between the two approaches. In the present clinical implementation, tensor-based deterministic tractography relies on a single principal diffusion direction per voxel and propagates streamlines along the dominant eigenvector. While this approach is computationally efficient and intuitive, it is particularly vulnerable to failure in regions of complex fiber architecture, such as areas of fiber crossing, fanning, or edema-induced diffusion alteration, conditions frequently encountered in peritumoral regions. As a result, deterministic algorithms may prematurely terminate tracts or deviate from their true anatomical course, leading to underrepresentation of the CST and an apparent increase in distance from stimulation-positive sites. Probabilistic tractography, on the other hand, explicitly models uncertainty in fiber orientation by sampling multiple possible directions within each voxel (25). This allows for a more comprehensive exploration of potential pathways, making it better suited to handle distorted diffusion profiles caused by tumor infiltration, vasogenic edema, or mass effect (26). In the present series, this methodological robustness translated into reconstructions that more closely matched intraoperative functional data, reinforcing the practical value of probabilistic approaches in surgical planning for motor-eloquent lesions.

Importantly, our findings are consistent with prior experimental and clinical studies suggesting superior sensitivity of probabilistic tractography in detecting functionally relevant white matter pathways (14, 25, 27–29). However, many previous reports relied on indirect validation methods, such as postoperative deficits or qualitative intraoperative impressions. The strength of the present study lies in the direct, point-by-point correlation between preoperative tractography and intraoperative DES, providing a quantitative and clinically meaningful comparison.

From a surgical standpoint, the tendency of deterministic tractography to overestimate CST distance may have tangible consequences. Overestimation could encourage more aggressive resections under the assumption of a wider safety margin, potentially increasing the risk of postoperative motor deficits. Conversely, probabilistic tractography, by offering a more conservative and functionally grounded estimate of CST proximity, may support safer decision-making during both preoperative planning and intraoperative navigation. The occurrence of postoperative hemiparesis in one patient with glioblastoma underscores the intrinsic vulnerability of motor pathways in infiltrative tumors and further highlights the need for the most accurate preoperative tools available.

Nevertheless, several limitations of this study must be acknowledged. First, the sample size is small, reflecting the strict inclusion criteria and the requirement for complete imaging and intraoperative mapping data. While the number of stimulation sites partially mitigates this limitation, larger prospective studies are needed to confirm the robustness and generalizability of our findings. Second, all imaging was performed on a 1.5 Tesla scanner with a relatively limited number of diffusion directions. Although this reflects real-world clinical practice in many centers, higher field strengths and advanced diffusion models could further improve tract reconstruction accuracy. Third, the assumption of a linear 1:1 relationship between stimulation current and distance, although widely adopted, represents a simplification and may not account for variations in white matter conductivity, axonal density, or local microstructural alterations induced by tumor infiltration or peritumoral edema, all of which can contribute to interindividual variability in subcortical excitability and tissue conductivity. Another well-known limitation remains the brain-shift, which can compromise the spatial accuracy of neuronavigation. To reduce brain shift, head positioning was tailored case by case so that the craniotomy was oriented as uppermost as feasible relative to gravity, thereby limiting gravitational cortical subsidence into the surgical field. The head was fixed with lesion-specific mild rotation and flexion/extension to optimize exposure while avoiding unnecessary brain relaxation. In addition, the craniotomy was deliberately limited to the smallest size compatible with safe cortical and subcortical mapping, in order to reduce CSF loss and subsequent displacement. All patients included in this study were male, which represents a potential selection bias. However, this demographic uniformity ensures a highly homogeneous cohort, reducing inter-gender anatomical variability. Furthermore, to the best of our knowledge, there is no robust evidence in current literature suggesting significant sexual dimorphism in white matter microstructural anatomy or in the neurophysiological response to subcortical stimulation within the neuro-oncological context. Therefore, we believe this gender distribution does not undermine the validity or the generalizability of our findings regarding the accuracy of DTI-based tractography. Nevertheless, future studies with a more balanced gender distribution are warranted to confirm these observations across a broader population. An additional limitation, particularly relevant for probabilistic tractography, is the potential generation of spurious streamlines. While probabilistic approaches may better capture complex fiber configurations and improve sensitivity near distorted or infiltrated white matter, this may occur at the expense of specificity, especially when seed density is high or stopping criteria are permissive. In the present retrospective study, we did not quantify false-positive streamlines, because the analysis was designed around pointwise correlation between DES-positive sites and tract proximity rather than full-tract anatomical validation. This aspect should therefore be considered when interpreting the apparent superiority of the probabilistic approach. Future studies should incorporate dedicated metrics for false-positive burden and evaluate how seeding strategy and tract selection thresholds affect the balance between sensitivity and anatomical plausibility.

Despite these limitations, the present study provides compelling evidence favoring probabilistic over deterministic CST tractography in the context of motor-eloquent brain tumor surgery. Our results support the integration of probabilistic algorithms into routine preoperative planning workflows, particularly when operating near the corticospinal tract. Importantly, tractography, regardless of the algorithm used, should never be interpreted in isolation, but rather as a complementary tool to intraoperative neurophysiological monitoring.

## Conclusion

Probabilistic DTI-based tractography demonstrates superior anatomical and functional accuracy compared to deterministic methods when validated against intraoperative DES. Its adoption may enhance the safety of tumor resections involving motor pathways by providing a more reliable estimate of CST location. Future studies combining advanced diffusion models, larger patient cohorts, and prospective outcome analysis will be essential to further refine the role of tractography in functional neurosurgery.

## Data Availability

The datasets presented in this article are not readily available because The dataset is restricted to academic use only. Requests to access the datasets should be directed to roberto.altieri@unicampania.it.
